# Giant mesenteric hemangioma of cavernous and venous mixed type: a rare case report

**DOI:** 10.1186/1471-2482-13-50

**Published:** 2013-10-30

**Authors:** Guang-Zhi Yang, Jing Li, Hua Jin

**Affiliations:** 1 Department of Pathology, The General Hospital of Beijing Military Command, Beijing 100700, China; 2 Department of Pathology, The 263rd Hospital of PLA, Beijing 101149, China

**Keywords:** Mesentery, Hemangioma, Cavernous and venous mixed type, Ileus

## Abstract

**Background:**

Although vascular tumours are one of the most common soft tissue neoplasms, those occurring in the gastrointestinal system are rare and cases involving mesentery are even further rare. Herein, we reported a rare case of giant hemangioma in mesentery of the small bowel.

**Case presentation:**

A 5-year-old girl was admitted to the emergency room with abdominal pain and vomit for two days. Ultrasonography and computed tomography showed a giant solid-cystic abdominal mass, preferring diagnosis of teratoma. A large neoplasm in the mesentery of the small bowel was found in the surgical exploration, which was then resected with the partial bowel. A brown honeycomb mass in size 16 cm×8 cm×5 cm was observed to adhere to the small bowel, and diagnosed as hemangioma of cavernous and venous mixed type in final pathology.

**Conclusion:**

The mesenteric hemangioma is extremely rare and the variable imaging tests are non-specific, thus the diagnosis is rarely made before surgery and usually established by histopathological investigation after surgery. So the mesenteric hemangioma is supposed to be differentiated in abdominal mass, either in adults or children. Complete surgical resection is the optimal treatment.

## Background

Vascular tumours are one of the most common soft tissue neoplasms which account for about 7 per cent of all benign tumours [1]. They occur widely in many organs, and most frequently in skin, mucosa, liver, central nervous system, and so on. Hemangiomas of the gastrointestinal system are rare, and scale for only 0.05 per cent of the tumours in intestine [2]. Hemangiomas involving mesentery are even rare, and there were approximately twenty case reports and only four of them were presented as big neoplasms in the English literature to our knowledge [3-6]. The four cases, without exception, were all adults. In previous reports, the intestinal and mesenteric hemangiomas were of capillary or cavernous type in histopathology [7]. Herein, we report one case of large mesenteric hemangioma of cavernous and venous mixed type in a 5-year-old girl who presented with incomplete ileus.

## Case presentation

**Figure 1:**
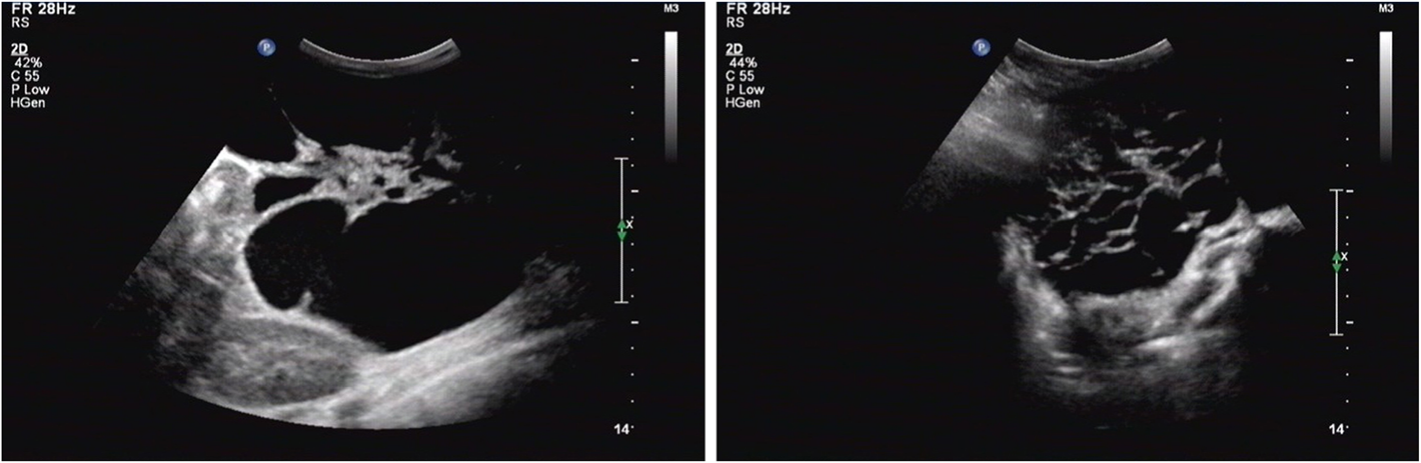
Ultrasonography displayed several echogenic areas in strip or tubal form communicating with each other in the lower abdomen and internal spotted medium echoes.

**Figure 2:**
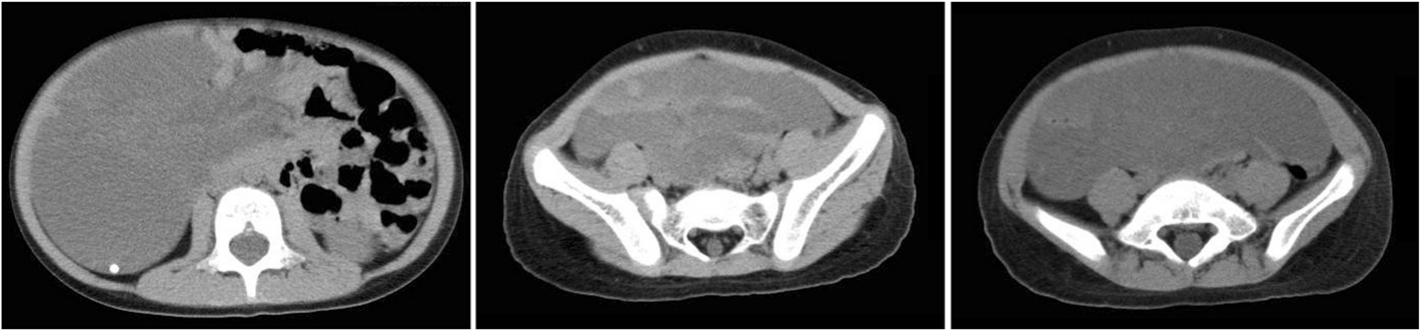
CT demonstrated a giant solid-cystic abdominal mass with heterogeneous density, which was mainly composed of cystic elements.

A 5-year-old girl was admitted to the emergency room with abdominal pain and vomit for two days. She had experienced of such symptom about twenty days ago and had naturally remited after fast. She had no history of anaemia and bloody stool. B-mode ultrasonography displayed several echogenic areas in strip or tubal form communicating with each other in the abdomen with the biggest diameter of about 6 cm and internal spotted medium echoes (Figure 1). Computed tomography (CT) demonstrated a giant solid-cystic abdominal mass with heterogeneous density, which was mainly composed of cystic elements (Figure 2). Given the age and sex of the patient, the diagnosis of teratoma was preferred. Clinically incomplete ileus was diagnosed and surgical exploration was performed immediately. During the laparotomy, a large neoplasm was observed in the mesentery of the small bowel, and the partial bowel combined with the neoplasm was resected. The symptom disappeared after operation and follow-up for three years was of no recurrence.

**Figure 3:**
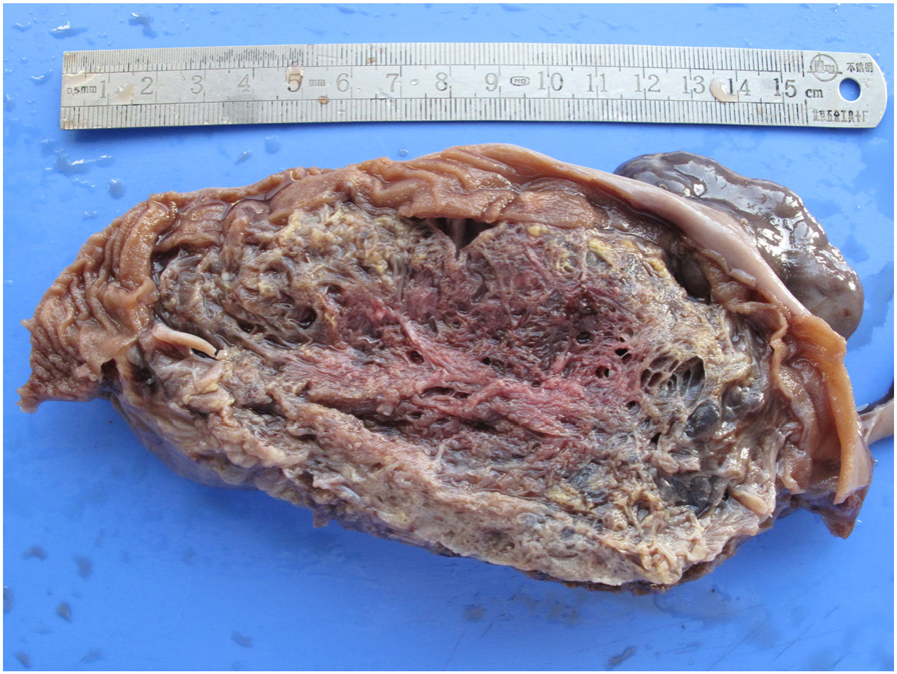
The giant hemangioma was adhered to the bowel wall, of which cut surface was of honeycomb.

**Figure 4:**
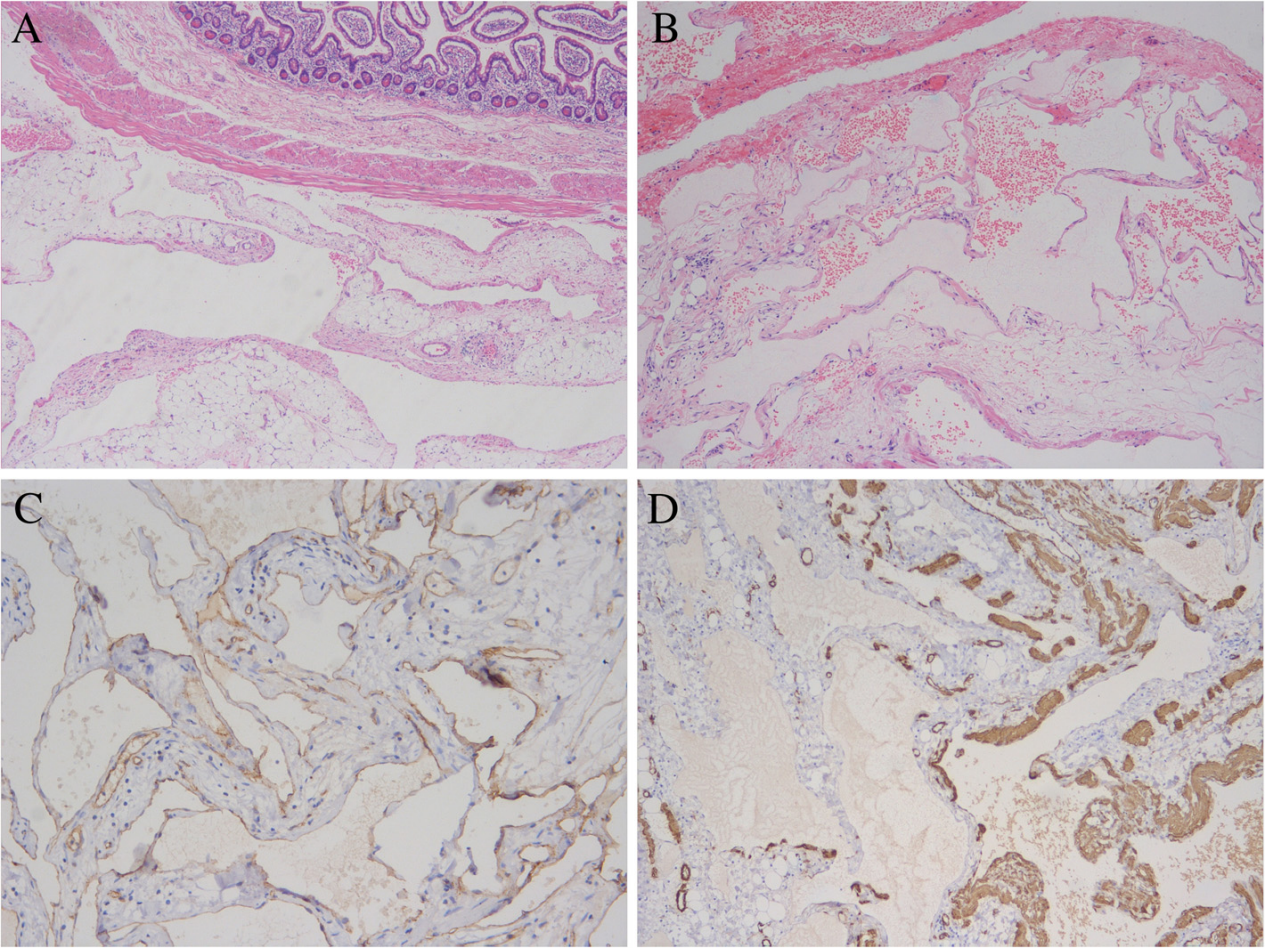
**The morphology and immunohistochemistry confirmed the diagnosis of hemangioma of cavernous and venous mixed type in mesentery. A**. The hemangioma was located in the mesentery and the small bowel wall was reserved. **B**. The tumour was characterized by diluted vessels, which most was with thin wall and minor was with thick wall. **C**. CD31 was positive in the endothelial cells flooring the vessels. **D**. SMA was positive in some vessels with thick wall.

Gross examination showed a brown honeycomb mass in size 16 cm×8 cm×5 cm, which adhered to the small bowel (Figure 3). Histological investigation revealed that the neoplasm was located in the mesentery and the bowel wall was not involved (Figure 4). Most parts of the neoplasm were composed of diluted vessels with thin wall, which was characteristic of cavernous hemangioma, whereas in other parts there were thick-walled vessels with less organized smooth muscles, which was characteristic of venous hemangioma. Immunohistochemistry for CD31, FVIII, D2-40, SMA was performed. CD31 and FVIII, biomarkers of blood vessel endothelia, were positive, while D2-40, biomarker of lymphatic endothelia, was negative. The element of venous hemangioma was confirmed with positive SMA in some blood vessels. Thus the diagnosis of mesenteric hemangioma of cavernous and venous mixed type was made.

## Discussion

Hemangioma is one of the most common benign tumours widely found in many organs, with a high occurrence during infancy and childhood. About 250 cases have been reported in the gastrointestinal system in the English papers since 1839. Hemangioma involving mesentery is extremely rare, and only twenty cases have been reported, among which four cases were in large size. The four cases were all adults, and no such patients of children or adolescents have been reported to our knowledge. Herein we reported a case aged 5 years in which the tumour was limited to the mesentery of the small bowel.

In histopathology, hemangioma may be classified into several categories according to vessel size and wall thickness [1]. Actually, not all types have been reported to occur in the gastrointestinal system. The classification of the intestinal hemangioma adopted by Abrahamson and Shandling was also supposed to be applied in mesenteric hemangioma [7]. The three types were capillary type, cavernous type, capillary and cavernous mixed type respectively, among which the most common one was cavernous type characteristic of diluted vessel with thin wall. In our case, the tumour was combined with two types. The majority was cavernous hemangioma, with minor part being venous hemangioma characteristic of diluted vessels with thick wall composed of less organized smooth muscles, which was also confirmed by immunohistochemistry. So it was of cavernous and venous mixed type in histology. To our knowledge, such hemangioma has been seldom reported previously except in the case of Ruiz and Ginsberg, in which some vessels had venous-like bundles of mural smooth muscle [3]. It is inferred that the type was not included in Abrahamson and Shandling’s classification due to its rarity.

Hemangiomas of mixed cavernous and venous type are supposed to be one variation of venous type by some pathologists because venous hemangiomas may also have area indistinguishable from cavernous hemangiomas [1]. However, in our case, cavernous hemangioma made up the most of the tumour, whereas the venous hemangioma only took up the minor part. So, the case was better designated of hemangioma of cavernous and venous mixed type. We observed that existence of veins in the tumour was also supposed to be one kind of vascular malformations.

The origin of mesenteric hemangioma is still uncertain. Most reported cases involved both bowel wall and mesentery. Given hemangiomas of mesentery are far rarer than those of bowel, they are always proposed to be originated from the bowel, especially submucosa [3]. But in some cases, including ours, the tumours were limited in the mesentery rather than the bowel wall, and it seemed that the hemangioma was originated from the mesentery.

The symptoms of intestinal and mesenteric hemangiomas were often bleeding or obstruction depending on its location [8-10]. In our case, the tumour was located in mesentery and the bowel wall structure is reserved, so no bleeding was observed. It mainly displayed mass effect thus resulted to ileus.

The preoperative diagnosis of mesenteric hemangioma was difficult, sometimes even nearly impossible. The imaging methods including B-mode ultrasonography, CT, and magnetic resonance (MR) and so on, although provide some useful information, only can demonstrate an abdominal mass rather than diagnose accurately [11-14]. The final diagnosis has to be made by resection and histopathological investigation.

The optimal treatment of intestinal or mesenteric hemangiomas is surgical resection. In our case, operation was adopted successfully. Radiation, cryotherapy and other therapies have been used for some non-resectable cases and more wildly cases named of hemangiomatosis, but the prospect was limited [3,15].

## Conclusion

We reported one rare case of giant mesenteric hemangioma, which was characterized of occurrence in a young child and pathology of cavernous and venous mixed type. The mesenteric hemangioma is extremely rare and the variable imaging tests are non-specific, thus the diagnosis is rarely made before surgery and usually made by histopathological investigation after surgery. The mesenteric hemangioma is supposed to be considered in differentiation in abdominal mass, either in adults or children. Complete surgical resection is the optimal treatment.

## Consent

Written informed consent was obtained from the guardian of the patient for publication of this Case report and any accompanying images. A copy of the written consent is available for review by the Editor-in-Chief of this journal.

## Abbreviations

SMA: Smooth muscle actin; MR: Magnetic resonance; CT: Computed tomography; cm: Centimetre.

## Competing interests

The authors declare that they have no competing interests.

## Authors’ contributions

GZ performed the pathological observation and collected the clinical information. JL made the pathological diagnosis and made up the manuscript. HJ performed the immunohistochemistry and participated in manuscript revision. All authors read and approved the final manuscript.

## Pre-publication history

The pre-publication history for this paper can be accessed here:

http://www.biomedcentral.com/1471-2482/13/50/prepub
